# Cookstove Smoke Impact on Ambient Air Quality and Probable Consequences for Human Health in Rural Locations of Southern Nepal

**DOI:** 10.3390/ijerph17020550

**Published:** 2020-01-15

**Authors:** Sagar Adhikari, Parth Sarathi Mahapatra, Chiranjibi Prasad Pokheral, Siva Praveen Puppala

**Affiliations:** 1 International Centre for Integrated Mountain Development (ICIMOD), G.P.O. Box 3226, Kathmandu 44700, Nepal; sagar.adhikari@icimod.org (S.A.); parth.mahapatra@icimod.org (P.S.M.); 2 National Trust for Nature Conservation (NTNC), Lalitpur 44700, Nepal; chiranpokharel@yahoo.com

**Keywords:** indoor/outdoor emission, emission factor, ambient air quality, health impact, AirQ+

## Abstract

Residential emission from traditional biomass cookstoves is a major source of indoor and outdoor air pollution in developing countries. However, exact quantification of the contribution of biomass cookstove emissions to outdoor air is still lacking. In order to address this gap, we designed a field study to estimate the emission factors of PM_2.5_ (particulate matter of less than 2.5 µ diameter) and BC (black carbon) indoors, from cookstove smoke using biomass fuel and with smoke escaping outdoors from the roof of the house. The field study was conducted in four randomly selected households in two rural locations of southern Nepal during April 2017. In addition, real-time measurement of ambient PM_2.5_ was performed for 20 days during the campaign in those two rural sites and one background location to quantify the contribution of cooking-related emissions to the ambient PM_2.5_. Emission factor estimates indicate that 66% of PM_2.5_ and 80% of BC emissions from biomass cookstoves directly escape into ambient air. During the cooking period, ambient PM_2.5_ concentrations in the rural sites were observed to be 37% higher than in the nearby background location. Based on the World Health Organization (WHO)’s AirQ+ model simulation, this 37% rise in ambient PM_2.5_ during cooking hours can lead to approximately 82 cases of annual premature deaths among the rural population of Chitwan district.

## 1. Introduction 

Exposure to polluted air has become a major concern in most of the low- and middle-income countries (LMIC) [1,2,3]. Globally, 91% of the total population is estimated to breathe polluted air while both household and ambient air pollution is responsible for seven million deaths in 2016 [1]. The World health organization (WHO, 2018) has also estimated that about three billion people are using polluting fuels, which are a primary source of household air pollution (HAP) [1]. Instead of using cleaner fuels such as liquefied petroleum gas (LPG), biogas, ethanol and electricity, the inefficient burning of solid fuels such as wood, coal, animal dung, charcoal, and agricultural residue generate high amount of smoke and hence, are polluting in nature and termed as polluting fuel [1]. In most of the LMIC, biomass fuel based cooking and heating is considered to be a significant source of household as well as ambient air pollution [2,3,4,5,6,7]. Previous studies estimated that residential biomass emissions make up more than 40% and 50% contribution to the PM_2.5_ (particulate matter less than 2.5 µ diameter) and black carbon (BC)/particulate organic matter (POM) emissions respectively in South Asia, East Asia, Eastern Europe, and East Africa [8]. Biomass combustion emits various gaseous and particulate pollutants that adversely affect human health [5,9,10,11]. A wide range of acute and chronic diseases are now associated with exposure to air pollution, starting from diabetes, declining cognitive functions, and chronic obstructive pulmonary disease (COPD), to cardiovascular diseases (CVD) [11,12,13,14] that lead to higher premature mortality and morbidity.

Black carbon is a constituent of PM and one of the byproducts of incomplete combustion of carbon-based fuels. Various studies have also indicated the adverse effects of BC on human health, climate and glacier retreat [15,16,17,18,19]. Bond et al. [18] reported that 20% of global BC emission are due to cookstove emission worldwide. A report published by WHO [20] found that the black carbon basically acts as a universal carrier of toxic chemicals, related to the inefficient combustion, of varying toxicity levels which are sensitive to the targeted human organ such as lungs, body’s defense cells, heart, etc. BC is one of the short-lived climate pollutants (SLCP) that has strong role in impacting climate change and glacier retreat [16,21]. The emissions of BC is correlated with other pollutants such as volatile organics, secondary organics, and poly-aromatic hydrocarbon, hence mitigation of BC can help to reduce other pollutants consequently [22,23,24].

Past studies on indoor/outdoor air quality examined the flow of pollutants between outdoor and indoor locations [1,11,12,17,18]. Infiltration of pollutants from the ambient air to indoor locations was reported in most of the developed countries, where the ratio of indoor to outdoor (I/O) pollutants (basically PM and BC) is less than one [25,26]. However, the opposite was found to be the case in most of the developing countries, where indoor air pollution is much higher than ambient air pollution, especially in rural areas, with I/O ratio greater than 1 and exfiltration of pollutants from indoors to outdoors was expected. In this context, only a handful of research has been carried out in different parts of the world by different researchers. Soneja et al. [27] conducted an experiment in a rural village of Nepal and estimated ~26% exfiltration of PM_2.5_ during biomass combustions. In another study conducted by McDonald et al. [28] reported that biomass-based heating and cooking activities contributed up to 80% of emissions in the ambient air of the residential areas in the USA. However, these studies were carried out using either a theoretical approach based on different physical and chemical models [27] or with hypothetical estimation rather than direct emission measurement both indoor and outdoor locations simultaneously. 

**Figure 1:**
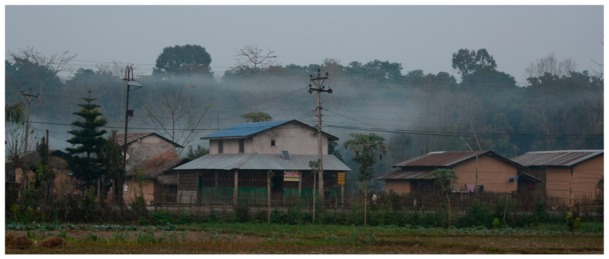
Smoke layer in a village due to cookstove emissions (Photo: Arnico K. Panday, ICIMOD; http://www.icimod.org/?q=35075).

**Figure 2:**
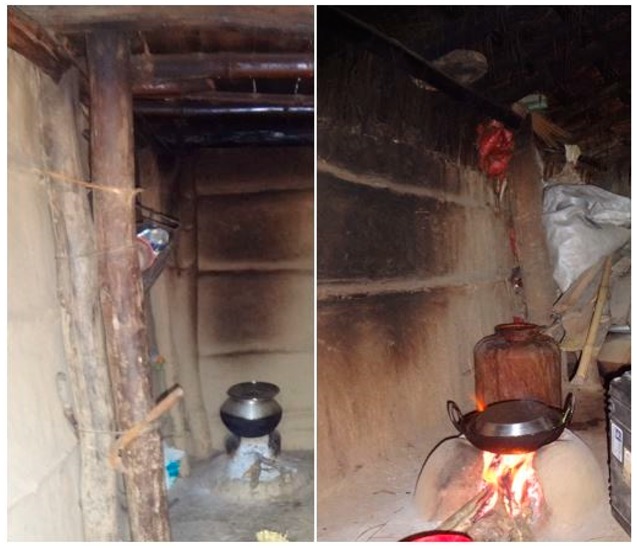
Picture showing black layers of smoke on the kitchen wall and ceiling.

In South Asia, higher levels of indoor air pollution are associated with biomass-based cooking and heating activities accompanied with poor ventilation. Simultaneously, formation of a thick smoke layer in the ambient air is commonly observed in villages at the time of cooking (Figure 1), indicating that a significant amount of smoke escapes from the indoor to the outdoor environment. Previous studies have also explained about the deposition of certain fraction of pollutants on the kitchen walls before exiting the kitchens to ambient environment [27,29]. Pictorial evidence of such deposition (wall and roof coated with black particles) can be found in Figure 2, indicating that in most of these rural households, certain fractions of particulates are trapped on the kitchen walls or roof instead of escaping outdoors. However, little information exists on what fraction of cooking emissions escapes into the ambient environment and its possible adverse health outcomes. Moreover, various tools/software such as AirQ, BenMap, AirQ+, etc. have been developed to understand the health impacts of air pollution [30,31]. Different air pollutants (PM_2.5_, PM_10_, NO_2_, and O_3_) can be used as input to these tools to derive acute and chronic health outcomes of air pollution exposure. However, these tools were developed based on outcomes of epidemiological studies in Western Europe and North America; hence using them in other regions of the world might involve uncertainties. 

This study aims to understand the health impacts of indoor biomass-based cookstove’s emission contribution to ambient air in rural settings of southern Nepal. To achieve this, we first estimated PM_2.5_ and BC emission factors of smoke near cookstoves (inside) and smoke escaping from the roof (outside). This information will provide the fraction of PM_2.5_ and BC emissions deposited on the walls/ceilings of rural households. Second, we measured ambient PM_2.5_ concentration in rural villages where biomass-based cooking is predominant [32] and compared this with a nearby background location without air pollution sources in the vicinity. This should provide the percentage of PM_2.5_ enhancement due to household emission’s contribution at the village level. Finally, we simulated the health impacts of village/background ambient air pollution and the contribution of cookstove smoke to ambient exposure by using AirQ+.

## 2. Study Site

**Figure 3:**
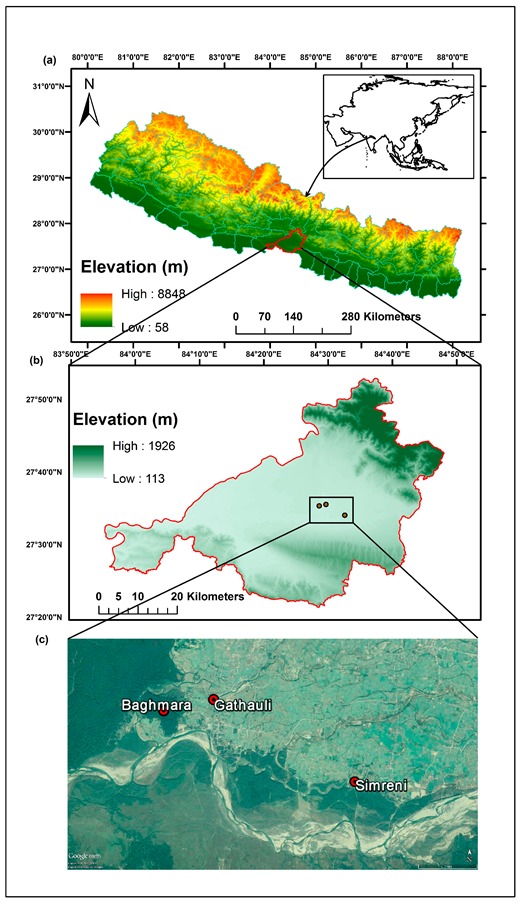
Figure showing (**a**) map of Nepal in the South Asia region, highlighting the study area in Chitwan district of Nepal; (**b**) enlarged map of Chitwan district showing the study area; and (**c**) Google Earth image showing study sites.

This study was conducted in selected rural locations of Ratnanagar municipality, Chitwan district in the southern plain region of Nepal adjacent to the Chitwan National Park (Figure 3). A recent report by the government of Nepal indicates that ~50% of households in Chitwan use biomass fuel for cooking and heating activities [32]. People in these villages primarily use biomass fuel to meet their daily energy requirements, making it a suitable location for undertaking the present study. Ambient air pollution measurements were conducted in three different sites (Figure 3), two from Gathauli and Simreni villages (photographs provided as Supplementary Material S1), and one from a background location in the forest area (Baghmara Community Forest). All the three locations were situated within an aerial distance of approximately 10 km and at similar elevation of 190 m. Each of these villages are comprised of 50–100 scattered households. The background location was chosen inside a dense community forest area with no anthropogenic source of emissions within 500 m of the sampling location. The same forest area also surrounds these villages from three sides. Hence, any changes in the pollutant concentration of the background location is also expected to show up at the village sites. However, as forests are considered pristine ecological zone [33], the background location in the present case would represent a cleaner micro-environment in comparison to the villages. Moreover, the background site also represents well-mixed air mass, both from regional and local sources of air pollution. We chose the summer season (April) for field measurements since indoor and outdoor heating is more common during the winter season in this part of Nepal which would introduce a bias in our measurement of cookstove smoke’s contribution to ambient air pollution. 

## 3. Materials and Methods

The fieldwork was carried out in two phases during April 2017. In the first phase ambient air pollution (PM_2.5_) measurements were taken from two villages and one background location. The measurements would show the difference between PM_2.5_ concentration at the village level and the background location during cooking and non-cooking time. The second phase of the experiment dealt with estimation of PM_2.5_ and BC emission factors from cookstove smoke measurements performed inside the kitchen and outside of the kitchen (primarily it is the highest plume emitting zone of the kitchen). This experiment quantified the difference between PM_2.5_ and BC emission factors of cookstove smoke inside the kitchen and the smoke spontaneously escaping out of the kitchen. Finally, the health impacts of cookstove emission’s contribution to ambient air pollution on the village population was quantified using AirQ+ model (World Health Organization, Copenhegen, Denmark) simulation.

### 3.1. Ambient Measurements

E-Samplers (Met One Instruments, Inc. Grants Pass, OR, USA) were used to measure the ambient PM_2.5_ concentration that siphoned air at a constant flow rate of 2 L/min (Supplementary S2). The E-Samplers were placed in a position that allowed free flow of air from every direction. Two E-Samplers were installed at both the rural sites and were mounted at a height of 3 m above the ground level near the village center. The third E-Sampler was installed at the Baghmara community forest area and placed on top of a watchtower built by the local forest department. E-Samplers were run for 20 days, and data were recorded at 5 min intervals. All E-Samplers used in the field study were co-located with a Grimm environmental dust monitor (EDM 180) running at Chitwan ambient air quality station for a few days before the campaign for any bias correction. Slope and intercept values obtained by comparing E-Samplers with the Grimm EDM were used for data correction. The E-Sampler has a relatively low-cost sensor for measuring PM and is based on the principle of measuring bulk aerosols light scatter, which might add bias in PM mass estimation [34]. Hence, values obtained from such a sensor were compared with that of the Grimm EDM 180, which has a high-quality and stable sensor based on the principle of measuring individual aerosol particle light scatter to provide high-quality measurements [35]. Data reported in this manner will have less uncertainty and be synchronized/calibrated with ambient conditions [36]. More details about the instrument inter-comparison and data correction are provided in Supplementary Material S2.

### 3.2. Inside-Outside Concentration Measurements

**Figure 4:**
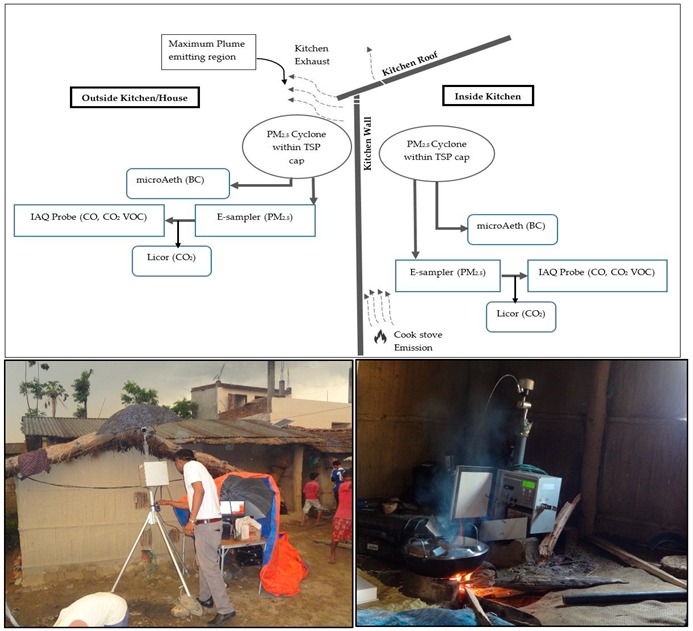
Top panel: schematics of the instrumental setup for cookstove emission measurement inside and outside the kitchen; Bottom right: indoor emission measurement; Bottom left: outdoor escaping emission measurement.

Emission factor estimates for different pollutants in cookstove smoke using biomass fuels were calculated inside and outside of the kitchen at four randomly selected households in the two villages where ambient measurements were being carried out. Sampling was carried out in one complete cooking cycle and no repeat measurements were performed. As we are estimating the emission factor of the pollutant per kilogram of fuel wood used, the evening and morning cooking might not affect the EF estimation. However, in this study we have also performed two morning and two evening samplings (Table 1). In order to perform the study a special setup was designed where the inlet of the E-Sampler was used to siphon air at a constant flow rate of 2 L/min for measuring the PM_2.5_ concentration. The outlet of this equipment was connected to the inlet of the indoor air quality (IAQ) probes (IQ-610, Gray Wolf Sensing Solutions, Ireland) for measuring the concentration of CO and CO_2_ from the same air mass (required to estimate the emission factors). An electrochemical sensor and Non-dispersive infrared (NDIR) sensors are used in the IAQ probe to measure CO and CO_2_ respectively. The microAeth, AE51 (AethLabs, CA) was also used to measure BC and its inlet was placed together with that of the E-Sampler in order to siphon the same air mass for all the measurements. The instrument is portable and siphons air at a constant flow rate of 50 mL/min. The inlet of the microAeth was fitted with a PM_2.5_ cyclone in order to prevent particles with a larger diameter (more than 2.5 micrometers) from getting inside the equipment. The schematics of the instrumental setup are illustrated in Figure 4. The internal time for all the equipment was synchronized and programmed to collect data at 1 min intervals. Inside the kitchen, the inlet of the E-Sampler was placed at a distance of ~1 m from the stove to sample the cookstove smoke plumes. This is a common condition practiced by various researchers in the past [23,27,37,38]. For the outside emission measurement, the inlet of the E-Sampler was placed in the immediate vicinity of the maximum visible smoke-escaping zone of the kitchen roof. Background measurements were also performed for both inside and outside locations to derive the background subtracted smoke concentration. The pollutant concentration before cooking events are the actual background concentration. The difference in the concentration between cooking and the background condition gives the actual concentration of cookstove smoke plume. This is common method for emission measurement and emission estimation [39,40,41]. Hence, the instruments were run at least 10 min prior to cooking for the background sampling and smoke was sampled from the complete cooking cycle from each of the household, which varied from 30 min to 1 h depending on the cooking tasks. The IAQ probe was calibrated with zero air and calibration gas mixtures (CO_2_, CO, and VOCs) before the field campaign. CO sensors were calibrated with 0, 5, and 95 ppm of standard CO gas supply whereas CO_2_ was calibrated at 0, 350, and 1250 ppm of standard CO_2_ gas supplies. BC data from both the microAeth were compared with BC data obtained from a high-quality aethalometer (AE33, Magee Scientific, CA, USA). Comparisons indicated a slope close to unity with R^2^ > 0.98. Hence, no corrections were applied to the BC measurements. Both the E-Samplers used for household and ambient measurement were also compared with the Grimm EDM 180 and applicable correction factors were used [35]. More details about the equipment, its comparison and response checks are provided in the Supplementary Material S2 and S3.

### 3.3. Kitchen and Fuel Composition

The details about the kitchen characteristics, cook stove type, fuel composition and other attributes related to cooking activity in the four households has been presented in Table 1. In all of the measured households, hardwood mixed with twigs were used for cooking indicating biomass as the primary fuel used in these households. We also conducted a questionnaire-based survey in 100 households in the study area. The survey was conducted to understand the households’ structure, general lifestyle, cooking pattern, fuel use, kitchen structure, etc. The information collected through the survey was used to select the four households representing the local context. The survey study revealed that around 90% of households relied on biomass fuel as their primary source of cooking fuel residing in poorly ventilated kitchen structures dominated by one pot traditional mud stove. These statistics are also corroborated by the Central Bureau of Statistics, Nepal [32] more than two-third of total population of Nepal residing in rural locations are using biomass fuel in traditional cookstove as their primary source of cooking fuel. A study conducted by Shrestha and Shrestha, on the ventilation and indoor air also revealed that 70% and 85% of households in flat land and rural locations of Nepal are poorly ventilated [42]. Hence, most of the smoke released from the stoves exited through the nearest roof-wall joints was observed during the field study period. Hence our measurement represents the dominant households of the region.

### 3.4. Emission Factor Calculation

The standard carbon mass balance method was used to compute the emission factor of the pollutants in this study [39,40,43]. A primary assumption for this method is that all carbon content in the fuel was emitted as carbon rich gases i.e., CO_2_, CO and total volatile organic compounds (TVOCs) along with carbon containing particulates such as black carbon and hydrocarbon. During the combustion of fuel, the majority of carbon fraction is converted into gaseous CO_2_ and CO along with minimal emissions of other carbonaceous species such as hydrocarbons (less than 4%) [44,45,46]. A study conducted by Ward and Hardy indicated that 95% of the total carbon released during a biomass combustion event is in the form of CO_2_ and CO emission [47]. Another study conducted by Mcdonald et al. [28] found that the average VOC emissions rate is 18% of the CO emission rate considering mixed hardwood and oak fuel combustion in wood stoves. Whereas, the same reduces to ~4% considering only mixed hardwood fuel. However, this comparison with CO_2_ emission rate might even yield in lower contribution of VOC’s. Hence, only the concentration of emitted CO_2_, CO and BC was taken as a proxy for combusted fuel [44,45,48,49,50]. Then the approximate emission factors of particulate pollutants were estimated from the ratio between pollutants and the emission factor of CO. Based on the above assumption, a standard equation [39,40] was used to obtain the emission factor of the pollutants. Details of the equation have been provided in Adhikari et al. [51]. The equation used for the calculation has also been provided in Supplementary S4. The carbon content of the wood sample was considered to be 50% as most of the studies conducted in the region have used this value to estimate the emission factor for fuel wood burning [39,40,41,49,52].

### 3.5. Air Q+

The AirQ+ model is a software tool developed by the World Health Organization’s (WHO) European center to estimate the health burden attributable to air pollution. [53,54]. The AirQ+ also helps to analyze the impacts on population in different emission scenarios [31]. Unlike other tools developed for the health risk assessments such as AirCounts, Aphekom, Economic Valuation of Air Pollution (EVA), SIM-Air etc, the AirQ+ can be used for any population size of the specified area with mortality and morbidity characteristics [55]. The Air Q+ model also gives the user options to run different scenarios to understand the health burden originating due to a particular air pollutant and for a particular type of disease in a given age group. Even the concentration response functions used for calculation of different health burdens by this tool have been well validated across different epidemiological studies [31]. Additionally, the Urban Health Initiative (UHI) program launched by the WHO in developing countries is supporting policy makers to understand the health effects and associated economic burdens of different pollutants. Within this initiative, Kathmandu in Nepal and Accra in Ghana were chosen as one of the first cities to implement the objectives of the UHI. In a series of workshops conducted by WHO in Nepal, the policy makers, health practitioners and researchers were made aware of the AirQ+ tool and explained its usage for decision making [56]. Hence, in the present study we made use of AirQ+ model considering its benefits and acceptability by the local policy makers. 

We used the WHO’s AirQ+ v 1.3 model to simulate the averted cases of mortality due to change in PM_2.5_ concentration at the village and background sites. Thereafter, the averted cases of mortality were also calculated for the rural location and background location with reference to Nepal’s Ambient Air Quality Standards (AAQS) for 24-h PM_2.5_ (40 µg/m^3^; in the absence of any annual standards) and WHO’s AAQS for the annual value of PM_2.5_ (10 µg/m^3^). A recent research performed by Conti et al. in 2018 [31] elaborately discusses how the Air Q model works and the improvements on the older version (AirQ) in the most recent version (AirQ+). Primarily, the health impact assessment performed using the AirQ+ model is based on the estimation of the attributable proportion (AP) [54]. This is defined as the portion of a particular health outcome (Ischemic Heart Diseases (IHD), Acute Lower Respiratory Infection (ALRI), Chronic Obstructive Pulmonary Disease (COPD)), in a well-defined population attributable to a given air pollutant. We also simulated the mortality due to IHD, COPD, and ALRI incidence in specific age groups. In order to determine the proportion of rural population affected by this pollution level, we subtracted the urban population (211,733) from the total population (579,984) of Chitwan during the year 2011 [57,58]. As census data is not available for the campaign year, i.e., 2017, we interpolated Chitwan’s rural population data from 2011 to 2017 by using the annual average population growth rate of Nepal (2.06%) [57,58]. We ran the AirQ+ model for different scenarios by using 2017 Nepal government data [59] on annual incidence of deaths per 100,000 people due to all causes (611.4), ALRI (31.1), IHD (100.45), and COPD (60.15).

## 4. Results and Discussion

### 4.1. Inside/Outside Cookstove Emissions 

The inside and outside cookstove smoke concentration along with emission factors of PM_2.5_ and BC (during the cooking event) for the four households are presented in Table 2. The average PM_2.5_ and BC concentrations of indoor smoke near the cookstove were found to be nearly 2–5 times higher than outside cookstove smoke concentrations. However, inside/outside CO and CO_2_ concentrations had less variation compared to PM_2.5_ and BC. This was mainly due to the deposition of certain fractions of particulate pollutants emitted from cookstoves (Figure 2). The dilution of pollutants will lead to a similar concentration ratio among different pollutants. However, variable CO/BC and CO/PM_2.5_ ratios indicate that a certain portion of PM_2.5_ and BC was lost during transportation from near the cookstove to ambient conditions. Additionally, the higher percentage loss of PM_2.5_ in comparison to BC can be attributed to their chemical constituents. Chemically, the particulate matter originating from biomass combustion include organics, inorganics, ions, minerals etc. [60]. Most of the PM_2.5_ may also consist of semi-volatile aerosols such as organic carbon, which are converted from gas to particle phase and may be in liquid form. Such aerosols may have a higher removal rate (deposition) due to their hygroscopic nature and liquid form [40,61]. Snider et al. also reported the zero hygroscopicity for BC while PM with its organic constituents, ions, inorganic constituents would exhibit a certain range of hygroscopicity [60]. This might lead to higher adhesion, deposition of PM particles compared to BC. This portion might represent the amount of PM_2.5_ and BC deposited on the walls or ceiling of the kitchen. As smoke is emitted from the cookstove, it gets mixed with the kitchen’s background air and gets diluted. Some fraction of aerosol particles settles down; some fraction gets deposited on the ceiling and roof structure of the kitchen and the rest exits from the vents and cracks of the walls and roofs. The deposited aerosol mass inside the house can be seen as a black coating on the walls and ceiling (Figure 2). The average PM_2.5_ concentrations of cookstove smoke varied between 1.5 and 9.5 mg/m^3^ for inside compared to 0.7 and 3 mg/m^3^ for outside; whereas the average BC concentration of cookstove smoke was observed to be in the range of 0.5 to 1.6 mg/m^3^ for inside compared to 0.3 to 0.5 mg/m^3^ for outside. Our observed concentration range aligns with the results of other field studies on indoor exposure or cookstove emission [38,39,62,63]. 

The inter-variability in PM_2.5_ concentrations of cookstove smoke among the households can be attributed to factors such as types of biomass fuel, kitchen structures/size/volume, cooking style, exfiltration rate, air exchange rate between inside and outside the kitchen, combustion temperature, moisture content of the fuel, carbon content of the fuel, and prevailing metrological conditions [27,40,64]. The BC/PM ratio varied between 0.15 and 0.31, indicating that around 20% of PM_2.5_ emissions represent BC. The variability in the BC/PM ratio can be attributed to different fuel combustion conditions. The smoldering condition, where fuel combustion temperature is lower than 225 °C, can lead to a high concentration of organic matter emission, which is major fraction of PM_2.5_ [65]. On the other hand, the flaming condition, where fuel combustion temperature is more than 300 °C, can lead to more elemental carbon (or BC) emissions [41,66]. In the case of BC, emission occurs in particulate form, whereas organic carbon is emitted in the form of volatile gases and mostly depends on gas to particle conversion. This intrinsic difference between fuel combustion and particulate formation can lead to variability of BC/PM_2.5_ ratios.

As mentioned above, decrease in the concentration levels of PM_2.5_ and BC is due to both dilution and deposition. We used emission factor calculation to determine what fractions of PM_2.5_ and BC were deposited on the kitchen walls and ceiling. Emission factor calculation of a pollutant is derived from the observed ratio of CO to the pollutant, where the dilution effect will be nullified. Along with other pollutants, CO is also diluted, thus the change in CO to the pollutant ratio can provide the amount of pollutant loss during transportation from the kitchen to ambient conditions. The emission factor of CO for inside the kitchens varied from 68 g/kg to 135 g/kg and was found to be almost similar to outside concentrations (Table 2). The average emission factor of PM_2.5_ for inside the kitchen was found to be 15.3 ± 7.9 g/kg whereas that for outside the kitchen was found to be 9.8 ± 4.9 g/kg of fuel. Similarly, the BC emission factors for inside and outside the kitchen were found to be 2.8 ± 1.2 g/kg and 2.2 ± 0.7 g/kg of fuel respectively. Overall approximately 35% of PM_2.5_ and 20% of BC emission were deposited or removed during transportation from the kitchen to ambient conditions. However, the variation in this percentage might be due to variability in the kitchen conditions such as kitchen volume, air exchange rate of the kitchen, presence of ventilation structures, partitions, wind movement etc. [64]. A similar study conducted by Soneja et al. using model and measurement estimates also reported that the exfiltration rate of PM increased by ~11% if the household had an open door or window. Hence, change in any of the factor might have contributed to the variability amongst individual kitchens. 

The estimated emission factor for both PM and BC was found to be slightly higher than in a few other studies [18,21,40,41,48] (Table 2). In most of the previous studies, the emission factors were estimated in a lab-based environment through a water-boiling test. During such tests, steady state combustions of fuel are maintained by controlling the fuel size, moisture content of the fuel, and air supplied to the fuel, and hence, might result in a lower emission factor of the pollutants [44]. However, another real-time measurement study for cookstove emission conducted during the NAMaSTE campaign in Nepal [40] estimated the emission factor of BC to be 0.221 ± 0.127 g/kg for hardwood cooking; while the emission factor of PM_2.5_ for different fuel types in a traditional cookstove ranged from 5.3 g/kg to 19.7 g/kg [39], with the average PM_2.5_ emission factor for hardwood of 10.66 ± 1.62 g/kg of fuel. Another field-based study conducted by Grieshop et al. [67] in India have also estimated the emission factor to be in the range of 1.7–24.19 g/kg for traditional cookstoves. Hence the emission factor estimation from the current study can be agreed against previous findings.

In the present study we calculated modified combustion efficiency (MCE) to interpret the different stages of combustion [40,51]. MCE is the ratio of carbon dioxide (CO_2_) released to the sum of carbon monoxide (CO) and CO_2_. This can be used as an indicator of flaming and smoldering phase of combustion [40,41]. Akagi et al. [41] mentioned that an MCE value near to 0.99 indicates relatively pure flaming condition, values less than 0.9 indicates smoldering and value around 0.9–0.95 indicates the combination of smoldering and flaming condition (mixed phase). According to the same study, during the flaming phase (high MCE), there will be high CO_2_ emission as ideally all fuel is being burnt efficiently. However, during the smoldering phase, indicated by a lower MCE, there is incomplete combustion of the fuel that results in higher emission of PM_2.5_, BC, organic carbon emission and other pollutants [41,68]. The average MCE value for all the households in the present study was estimated as 0.91, and details of individual households have been mentioned in Table 2. The real-time emission factor and MCE plot for all the households shows variability in both PM and BC emission factors during the smoldering and flaming phase of combustion, corroborating the above statement (shown in Supplementary Material S5).

In some studies, rather than directly measuring the concentration of the pollutants, it was estimated using the decaying chemical regression model and other chemical models. A study based on ventilation and particle deposition rate, conducted by Venkataraman et al. [5] indicates that 80% of the emissions escape from indoors to outdoors in households using biomass-based cookstoves. Though this was a hypothetical estimate, the emission contribution from indoor to outdoor was almost similar to our current estimates. A study conducted in the rural village of Pakistan [3] found that the ratio of indoor PM_2.5_ to outdoor PM_2.5_ is 4.37, indicating a high indoor PM_2.5_ concentration. This study also revealed that both indoor and outdoor PM_2.5_ concentrations are higher during the cooking period. Soneja et al. [27] used the concentration decay regression model to estimate the PM_2.5_ exfiltration ratio for 50 households and estimated the average PM_2.5_ exfiltration of 26% (95% CI; 22.4%–30.5%) ranging from 5.8% to 57.6%. Our observation results (66%) is higher than this model based estimate which may be due to the variability in kitchen designs and real life measurement conditions. Soneja et al. conducted the experiment in a constructed mock house and in occupied houses, making the condition similar in both the locations. However, in our study only four houses with real life working conditions has been taken for emission measurement study. The experiments were conducted under normal conditions without constraining any variable in order to simulate the real-life emission scenario. Hence, this might have attributed to the changes in emission estimate from that of the present study in comparison to that by Soneja et al. [27].

**Table 2:** Particulate matter of less than 2.5 µ diameter (PM_2.5_) and black carbon (BC) emission concentration in (µg/m^3^), CO, and CO_2_ emission concentration in (ppm) with emission factor (g/kg) and inside to outside contribution in percentage based on emission factor of pollutant.

Kitchen	Concentration (µg/m3)				Concentration (ppm)				Emission Factor (g/kg)							MCE	Out/In (%) Contribution	
	In		Out		In		Out		In				Out					
	PM_2.5_	BC	PM_2.5_	BC	CO	CO_2_	CO	CO_2_	CO_2_	CO	PM_2.5_	BC	CO	PM_2.5_	BC		PM_2.5_	BC
1	1527	476	772	314	36	568	26	422	1716	68	6.2	1.9	66	4.2	1.7	0.94	68	89
2	6753	1213	1691	376	75	563	32	244	1580	135	25.4	4.6	133	14.8	3.3	0.88	58	72
3	4513	707	3083	493	69	708	51	529	1647	102	14.1	2.2	10	12.9	2.1	0.91	92	93
4	9533	1616	2115	514	132	1372	57	669	1657	101	15.4	2.6	91	7.1	1.7	0.91	46	66
Average	**5581**	**1003**	**1915**	**424**	**78**	**803**	**41**	**466**	**1650**	**101**	**15.3**	**2.8**	**97**	**9.8**	**2.2**	**0.91**	**66**	**80**
Previous studies															Location			
Kurmi et al. [62]	5648	-									-	-			Nepal (*n* = 24)			
Chen et al. [38]	3469	-									-	-			Nepal (*n* = 2980)			
Jayarathne et al. [39]	9027	-									-	-			Nepal (*n* = 18) (Lab)			
Sota et al. [63]	10,563	-									-	-			Senegal (*n* = 8)			
Roden and Bond [44]	-	-									4.9–16.1	0.2–3.4			Hondurus (*n* = 12)			
Venkataraman et al. [69]	-	-									-	0.38–0.62			India (*n* = 4)			
Street et al. [21]	-	-									3.90	-			Estimation			
Akagi et al. [41]	-	-									6.64	0.83			Compilation			
Stockwell et al. [40]	-	-									0.22	-			Nepal (*n* = 2)			
Jayarathne et al. [39]	12,877	677.9									10.66	-			Nepal (*n* = 2)			
Grieshop et al. [67]	-	-									1.70	0.24–2.4			India (*n* = 26)			
Guofeng et al. [43]	-	-									2.2	0.83			China (*n* = 17)			
Dasch [70]	-	-									10	-			India (*n* = 18)			
Saud et al. [71]	-	-									4.34	-			India (*n* = 18)			
Saud et al. [29]	-	-									-	0.35			India (*n* = 17)			

‘*n*’ is the sample number.

### 4.2. Ambient PM_2.5_ Variation with Meteorology

The average ambient PM_2.5_ concentration in the two villages for 20 days was found to be 98.43 ± 47.17 µg/m^3^ and that for the background site was 84.47 ± 28.73 (Table 2). The inter-comparison of PM_2.5_ level between the two villages (Simreni and Gathauli) showed approximately 10% higher PM_2.5_ concentrations in Simreni during the entire measurement period (Supplementary Material S6). The inter variability of the PM_2.5_ level between these two villages might be attributable to the number of households in each village. A higher number of households in Simreni would consume more biomass fuel and consequently higher pollution levels are expected in comparison to Gathauli. The measured ambient PM_2.5_ concentrations for both villages and background location were compared and analyzed with the meteorological parameters. The average temperature, pressure, and wind speed at the background and village locations were found to be similar (Supplementary S7). The average ambient temperature of the village and background locations were found to be 27.6 and 26.1 °C respectively. In both locations calm winds were observed during the sampling period (average wind speed ~0.7 m/s) which indicates that meteorological changes did not play a significant role in PM_2.5_ variation. Rather a regional forest fire event raised the concentrations of PM_2.5_ for a particular period (10 April to 15 April) which was same for the village and background locations. The regional pollution level increment due to forest fire were evident by National Aeronautics and Space Administration’s (NASA) fire maps captured through Moderate Resolution Imaging Spectroradiometer (MODIS) satellite. The fire maps for 4 different dates has been provided as Supplementary Material S8. The first map on 4th of April shows the region around the sampling location doesn’t have any open fire event. On the 8th of April, very few forest fire events were captured, but on the 12th of April huge events had been captured throughout the region which would be the primary cause of increment of PM level during these days. Eventually, the fire events were reduced as the map on the 15th of April did not capture the fire events around the sampling locations and the level of PM_2.5_ also lowered. A recent study by Mehra et al. [72] also described similar trend of increment due to forest fire events in April 2016 in Chitwan. However, the forest fire events are not expected to affect our sampling frame as it is a regional event and equally affecting the village and background locations. 

### 4.3. Estimating the Effect of Cookstove 

**Figure 5:**
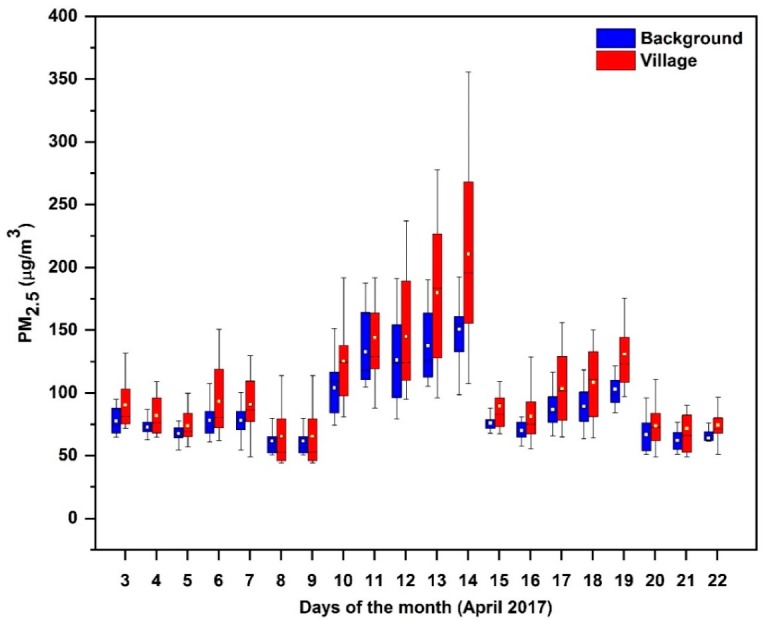
Box plot showing daily variation of PM_2.5_ (µg/m^3^) concentration for background (blue shade) and village (red shade). The upper and lower edge of each box represent the 75th and 25th percentiles, respectively; the top and bottom of the whisker represent the 90th and 10th percentiles, respectively. The square box mark within each box represents the average PM_2.5_ concentration and the horizontal mid-line in each box represents the median.

During the entire measurement period, the average PM_2.5_ concentration in the villages was observed to be higher than the background concentration. On an average the village PM_2.5_ concentration was found to be 16% more than background level concentration. Both the village and background 24 h average PM_2.5_ concentrations were found to be more than the WHO guideline of 25 µg/m^3^ and the national ambient air quality standard (NAAQS) of Nepal (40 µg/m^3^). A box plot showing PM_2.5_ concentration variation in 20 days for both the village and background locations is presented in Figure 5. Zhou et al.’s [73] research in west Africa found that 74%–87% of PM_2.5_ in the village area were due to biomass burning cooking activities. Another study conducted in Mexico by Watson and Chow [74] reported that nearby sources contribute almost 12% elemental carbon in the ambient air. Chowdhury et al. [7] found that biomass combustion contributed 7%–20% of total PM_2.5_ in ambient air in different cities in India. Chafe et al. [6] also estimated that household cooking with solid fuel contributed 12% of PM_2.5_ (APM_2.5_ population weighted PM_2.5_) globally. During the field campaign, villages were identified as one of the major sources of emission from biomass cookstoves and occasional household garbage burning; this indicated that the difference between background location and village level ambient PM_2.5_ concentrations was due to household emissions. 

**Figure 6:**
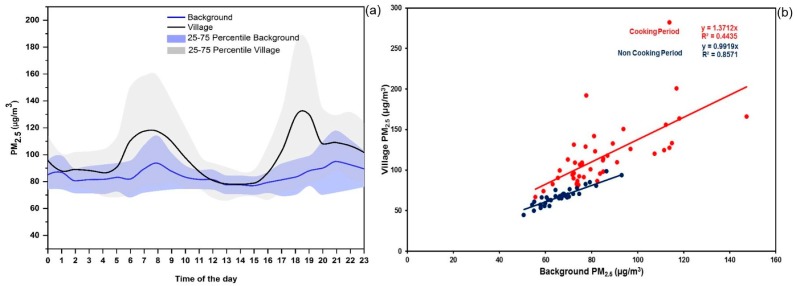
(**a**) Plot showing diurnal variation of PM_2.5_ concentration (µg/m^3^) for background (blue line) and semi-rural site (black line). The light blue shade and light gray shade represent the 25th and 75th percentiles of background and semi-rural sites concentration respectively. (**b**) Scatter plot between semi-rural sites and background level concentration during both cooking and non-cooking hours. Blue dots represent the non-cooking hour and red dots represent the cooking hours with linear fit, with blue and red line respectively for non-cooking and cooking hours.

In order to identify the relative contribution of biomass smoke to ambient air at the village level, we compared the mean PM_2.5_ diurnal cycles of the two rural village sites with that of the background location (Figure 6). Both the diurnal variation plots indicated the well-known morning and evening peaks typical to any urban/rural/semi-rural sites [75,76]. Such peaks occur pertaining to the changes in the boundary layer height which are completely different from the cook stove smoke emission peaks. Generally, the PM_2.5_ concentration starts rising after 05:00 h local time (LT) and reaches a maximum at around 07:00–08:00 h LT and gradually starts declining due to the changes in the nocturnal boundary layer. However, during the daytime, the boundary layer is highly expanded due to increase in temperature thus maintaining a lower PM concentration and well mixed atmospheric condition. Again PM_2.5_ level starts rising after 15:00 h reaching a maximum at around 18:30–19:30 h LT and then reduces. Amidst this boundary layer rise and fall, maximum cooking activity is observed within 06:30–0830 and 18:30–20:30 h LT which is strongly observed by the increase in the diurnal PM activity of the village in comparison to the background site. Since there is no vehicular movement or transportation activity in those villages during these hours, road dust contribution or vehicular emissions can be ignored. Additionally, these villages are situated far from the highway, bus stops or any public transport pathways. The background and rural site’s PM_2.5_ concentration was found to be almost similar between 11:00 and 15:00 h LT, indicating a well-mixed atmosphere. As per the questionnaire-based survey (discussed in Section 3.3) and previous research undertaken in this region [16], cooking period in the majority of household ranged from 06:30 to 08:30 h and 18:30 hr to 20:30 h LT. A comparison between villages and the background location during cooking and non-cooking hours (considering 1230 to 1430 h LT) is presented in Table 3.

The avergae village level and background level PM_2.5_ concentration during cooking hours was found to be 116.94 ± 38.97 µg/m^3^ and 84.94 ± 18.96 µg/m^3^ respectively. The additional ~32 µg/m^3^ of PM_2.5_ was mainly contributed by cooking activity during the summer season. Considering the individual village level concentrations, Simreni’s PM_2.5_ level is approximately 10% higher than Gathauli’s concentration during cooking hours. The PM_2.5_ concentration during non-cooking hours for village and background were 67.64 ± 10.48 µg/m^3^ and 66.85 ± 8.58 µg/m^3^ respectively. We also constructed a scatter plot between cooking and non-cooking hours PM_2.5_ concentration of the rural and background locations. The cooking period showed an enhancement of ~37% at the village site compared to the background site; while non-cooking hours had a similar PM_2.5_ level, indicating a well-mixed atmosphere and similar sources of emissions impacting all three sites during that period. These results also indicate that even though cookstove emissions influence ambient air quality, there is a significantly high background pollution in the region.

### 4.4. Health Risk Assessment Using Air Q+

As we observed in earlier sections, village level household emission significantly contributes to the ambient air quality. Hence, if we convert polluting household energy practices to clean energy such as LPG, biogas and electric, we can reduce village ambient pollution to background pollution levels. We estimated the health benefits of village level clean energy transition by using the AirQ+ model. Moreover, significant high background pollution was also observed during the field study, which may have its own health impact. Hence, we also quantified the health benefits of bringing down the background air pollution to WHO or Nepal’s national standards by using the AirQ+ model.

We agree that the PM_2.5_ data used in this simulation is for a shorter time period, while the calculations of the model use the data for an annual range, but this data would be representative of the overall pollution levels in that region. In this regard a recent study by Mehra et al. [72] performed over Chitwan during February to May 2016 observed average PM_2.5_ concentrations to be 95.9 ± 49.0 µg/m^3^ that more or less match the results of our study. Some cities in the nearby region situated south of the study site also reported similar values of annual PM_2.5_ concentrations, i.e., Delhi (128 ± 81 µg/m^3^ during January 2012 to December 2016) [77], Varanasi (92.5 ± 49.8 µg/m^3^ during March to December 2013) [78] and Kanpur (89.1 ± 62.1 µg/m^3^ during June 2015 to May 2016) [79], thus providing robust evidence that data obtained from our study can be used for understanding long-term health effects.

The model runs indicate that 82 cases of mortality due to all causes could be averted in Chitwan district if villages adopt cleaner technologies. These 82 cases of mortality reduction can be observed by reducing village ambient PM_2.5_ concentration from 98 µg/m^3^ to background concentration of 84 µg/m^3^ (Table 3). Relatively high background ambient PM_2.5_ concentrations were observed due to the location of the study site. The sampling location of southern Nepal is situated next to the Indo-Gangetic Plain (IGP), which is known to have a high population density, pollution sources and thick haze [80]. Several studies reported IGP air pollution and its widespread nature through observation, model and satellite based studies [81,82,83,84]. Hence, IGP pollution can be one of the major contributors to background pollution at the sampling site. Further, Chitwan district and its surrounding area has several small villages like that in the present study, where household emission is a major source of air pollution. These clusters of villages can also contribute significantly to the background pollution. Thus a fraction of this background pollution can be from local district level or IGP/regional scale.

**Figure 7:**
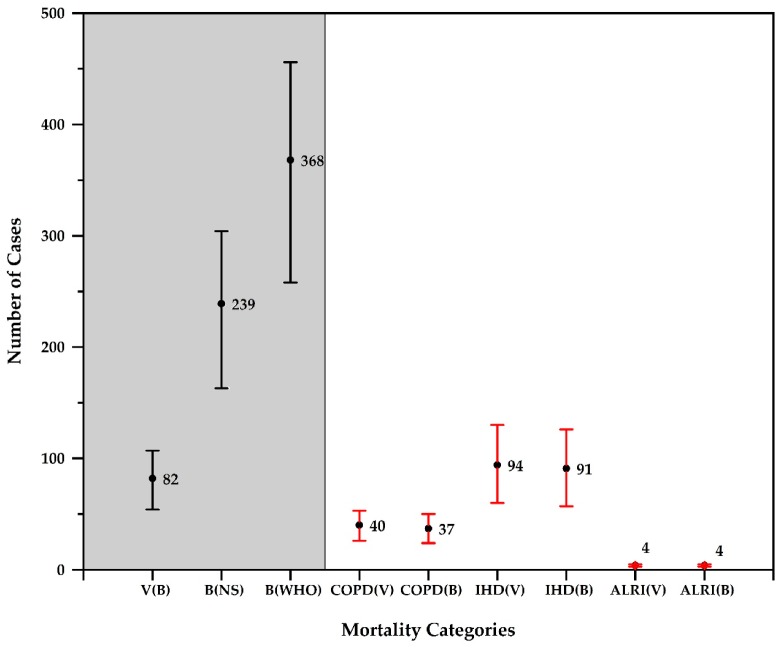
Premature mortality due to PM_2.5_ for different causes (with high and low range) during the study period. V (B), B (NS), B (WHO), COPD (V), COPD (B), IHD (V), IHD (B), ALRI (V), and ALRI (B) represent mortality averted (shaded portion) and mortality caused (non-shaded portion). Where, V(B) is mortality averted when village concentration lowered to background level. B(NS) is mortality averted when background concentration lowered to NAAQS of Nepal. B(WHO) is mortality averted when background concentration lowered to WHO standards. COPD(V) is mortality caused due to PM_2.5_ from background and village for chronic obstructive pulmonary disease. COPD(B) is mortality caused due to PM_2.5_ from background only for chronic obstructive pulmonary disease. IHD(V) is mortality caused due to PM_2.5_ from background and village for ischemic heart disease. IHD(B) is mortality caused due to PM_2.5_ from background only for ischemic heart disease. ALRI(V) is mortality caused due to PM_2.5_ from background and village for acute lower respiratory infection. ALRI(B) is mortality caused due to PM_2.5_ from background only for acute lower respiratory infection.

If observed background concentrations (84 µg/m^3^) (Table 3) could be reduced to Nepal’s 24-h standards (40 µg/m^3^) or WHO’s annual standards (10 µg/m^3^), approximately 239 and 368 deaths respectively could be averted. Current estimates indicate that switching to cleaner fuels can definitely increase health benefits, but reducing background pollution would increase such benefits three or fourfold. These results indicate the importance of background air pollution in the region and the need for large-scale air pollution control measures. With the present pollution levels for village concentrations, there could be 40 (26–53), 94 (60–130), and 4 (3–5) cases of mortality due to COPD, IHD, and ALRI respectively. Considering the background PM_2.5_ concentrations, there could be 37 (24–50), 91 (57–126), and 4 (3–5) cases of mortality due to COPD, IHD, and ALRI respectively (Figure 7). However, the difference in number of COPD, IHD, and ALRI cases between village and background air pollution is 3, 3, and 0, respectively, which also represents the village-level household contribution to ambient air pollution. This result indicates that most of the COPD, IHD, and ALRI cases could be attributed to background pollution. 

### 4.5. Limitation

The sample number for emission analysis is one of the limitations of the present study. It was difficult to get the house-owners’ approval for conducting real time study inside their kitchens, either due to personal, religious and cultural reasons or because of the confined space in the kitchen. However, the past several studies conducted in real households were also limited by the sample size and have explained in detail about the sampling difficulties. The sampling number in those studies conducted in various countries varies from as low as two to less than fifteen [39,40,63,64] suggesting the acceptance of smaller sample size for emission measurement studies. Moreover, the empirical relationship between the emission factors and the ambient pollutant concentration were not derived in the present study. The reason being, such an assessment requires model based analysis i.e. using simple box model. However, these models requires data for ventilation coefficient along with different meteorological parameters which are not available in the present study hence can be considered as a future scope of this study. 

## 5. Conclusions

In this study we estimated biomass fuel based cookstove emission factors for PM_2.5_ and BC for ambient conditions as 9.8 g/kg and 2.2 g/kg of fuel respectively and 15.3 g/kg, 2.8 g/kg for PM_2.5_ and BC respectively for indoor conditions. These emission factors represent real household conditions and accounts for deposition loses in the kitchen micro-environment. Approximately 35% of PM_2.5_ and 20% of BC emitted from cookstoves were deposited/decayed in the kitchen micro-environment. A higher percentage of PM_2.5_ loss can be attributed to its intrinsic properties. Nearly 20% of PM_2.5_ emissions represent BC directly from cookstoves; this percentage changed to ~30% in ambient conditions due to higher loss of PM_2.5_ in the kitchen micro-environment. Ambient air quality of rural villages was significantly influenced by biomass-based cooking activities. During the campaign period (summer season), cooking activity contributed 16% of ambient PM_2.5_ in rural locations on a daily basis; this contribution rose to 37% during the cooking hours. Regional and local background pollution is a major contributor to ambient air quality in the rural villages of Chitwan district, southern Nepal. Results of AirQ+ model simulations indicate that shifting cooking practices from solid fuel use to LPG or electricity at the village level could assist in averting 82 cases of premature mortality. Model results also indicate that background contribution of PM_2.5_ has a 3–4 times higher premature mortality from different causes while compared to the cooking related emissions. Hence, we demonstrated that the regional and background sources are the major ones while local sources such as biomass-based cooking activities also contribute significantly to health risk of rural population of Nepal. This will allow policy makers to understand the health benefits of shifting to clean energy at the country level and the adverse health effects of transboundary pollution.

## Figures and Tables

**Table 1:** Kitchen and fuel characteristics of the measured households.

HHS Number	Cookstove	Cookstove Materials	Fuel	Date	Cooking Time	Cooking Duration (Minutes)	Ventilation Status (Based on Observation)
**1**	Traditional one pot mud cookstove	Mud, stone	Biomass	03/04/2017	Evening	32	Medium (one door with one window)
**2**	Traditional one pot mud cookstove	Mud, stone	Biomass	04/04/2017	Morning	42	Poor (one door with no window)
**3**	Traditional one pot mud cookstove	Mud, stone	Biomass	04/04/2017	Evening	62	Medium (one door with one window)
**4**	Traditional one pot mud cookstove	Mud, stone	Biomass	05/04/2017	Morning	64	Poor (one door with no window)

**Table 3:** Comparison of PM_2.5_ level between village and background location in various periods.

Period	Background PM_2.5_ (µg/m^3^)	Village PM_2.5_ (µg/m^3^)	Average Exceedance (%)
Cooking	84.94 ± 18.96	116.94 ± 38.97	37.67
Noncooking	66.85 ± 8.58	67.64 ± 10.48	1.18
Twenty Days Average	84.47 ± 28.73	98.43 ± 47.17	16.53

## Data Availability

Data used for this study can be obtained by sending an email to Siva Praveen Puppala (SivaPraveen.Puppala@icimod.org).

## Supplementary Materials

The following are available online at https://www.mdpi.com/1660-4601/17/2/550/s1, Figure S1: Photograph of villages in the study area. Figure S2: Comparison of data from Grimm and E-Sampler (used for indoor measurement). Figure S3: Comparison between aethalometer and microAeth measurements of indoor (left) and outdoor (right) emissions. Figure S5: Figure showing real-time emission factor of PM_2.5_ and BC for all kitchens indoor and outdoor along with real time indoor MCE for Kitchen 1. Figure S6: Daily average plot of all three ambient sites. Figure S7: Real time measurement of (from top to bottom) ambient PM_2.5_ (µg/m^3^), temperature (°C), wind speed (m/s) and wind direction (degree) for all of the measured location. Figure S8: MODIS active fire images for (a) 3 April (b) 8 April (c) 12 April and (d) 15 April 2017. The yellow circles in each of the map covers the study site. Table S1: Correction factors of E-Samplers used for the study. Table S2: Correction factors of microAeth used for the study.
