# Supplementary material for: Cookstove Smoke Impact on Ambient Air Quality and Probable Consequences for Human Health in Rural Locations of Southern Nepal
Source: Int J Environ Res Public Health. 2020 Jan 15;17(2):550. doi: 10.3390/ijerph17020550 (PMC7014065; doi:10.3390/ijerph17020550)
Supplement: Supplementary file 1 [file ijerph-17-00550-s001.pdf]

Cookstove Smoke Impact on Ambient Air Quality and Probable Consequences for Human Health in Rural Locations of Sothern Nepal

Sagar Adhikari[1], Parth Sarathi Mahapatra[1], Chiranjibi P. Pokheral[2], Siva Praveen Puppala[1]*

[1] International Centre for Integrated Mountain Development (ICIMOD), G.P.O. Box 3226, Kathmandu, Nepal

[2] National Trust for Nature Conservation (NTNC), Laliltpur, Nepal

* Corresponding author's email address: SivaPraveen.Puppala@icimod.org

Supplementary Material

S1: Photographs of villages in the study area

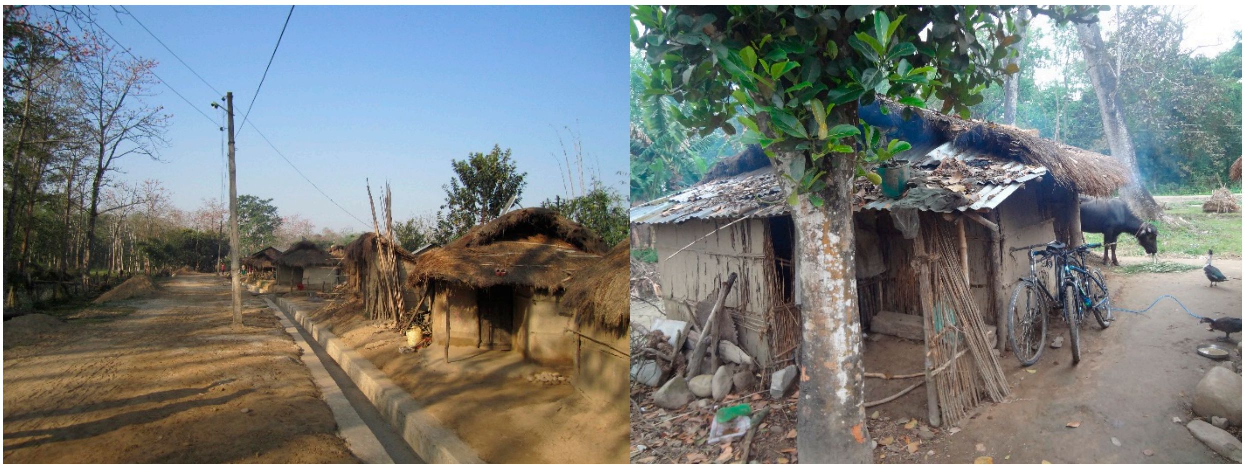

Figure S1: Photograph of villages in the study area

S2: Descriptions of E-Sampler and Grimm, comparison between E-Sampler and Grimm, and inter-comparison of E-Sampler between two villages

E-Sampler

An E-Sampler is a portable particulate matter measuring equipment manufactured by Met One Instruments, Inc., USA. Particulate concentration is measured with dual technology. It measures real-time particulate matter concentration along with filter collection, which enables us to measure the concentration by the gravimetric method. The E-Sampler works according to the principle of near-forward light scattering technique for bulk aerosols. The dust present in the air sample scatter light and is proportional to the particle load. The E-sampler is calibrated in the factory using polystyrene latex spheres of known index of refraction and diameter at multiple points to validate the linearity. Details about the equipment can also be found at https://metone.com/products/e-sampler/. The E-Sampler can be used to measure either PM_{10} or $PM_{2.5}$ based on the selected sharp-cut cyclone provided during the sampling. In the present study of ambient and cookstove emissions, we used the $PM_{2.5}$ sharp-cut cyclone. The E-Sampler has been used in many previous studies[1–4]. Results obtained from the E-Sampler have also been compared with other PM measuring instruments for better analysis [5].

Grimm

The environmental dust monitor (Grimm EDM 180) is a standard stationary optical aerosol-monitoring instrument designed by Grimm Aerosol Technik GmbH & Co. Kg, Dorfstrasse-9, Germany. It can simultaneously measure the mass fraction of PM_1, $PM_{2.5}$, PM_{10} and TSP in 0.1 μg/m^3 resolution. Particle concentration are measured according to the principle of light scattering of a single particle at a constant air sample flow rate of 1.2 L/min. To protect the semi volatile loss of particle during sampling, an isothermal inlet system was made that uses the Nafion dryer. The Grimm is widely used by the scientific community for atmospheric research [1,6–8]. In this study, a factory calibrated Grimm placed at Chitwan's air quality monitoring station (CAQMS) was used. Details about the instrument can be found at https://www.grimm-aerosol.com/products-en/environmental-dust-monitoring/approved-pm-monitor/edm180/.

Comparison between E-Sampler and Grimm

To correct the bias in reading of the E-Sampler, data obtained from it was compared with data obtained from the standard equipment, Grimm. A correction factor was derived by comparing the data and used to correct the E-Sampler data. Data from all five E-Samplers (two used in village ambient measurement, one used in background air measurement and two others used in indoor and outdoor measurement) were compared with data from the Grimm placed in CAQMS operating simultaneously. An example graph showing a comparison of data from the E-Sampler with data from the Grimm is presented in **Figure S2.** The variation in the observed concentration can be attributable the measurement process of the two instruments. Grimm is accompanied with Nefion dryer and thus avoiding any loss of volatile organics [9]. Mahapatra et al [1] also stated about the underestimation PM2.5 by esampler due to loss of volatiles during the measurement compare to Grimm. Hence the difference in the concentration is expected between Grimm and Esampler. The correction factors of all other E-Samplers are given in **Table S1.**

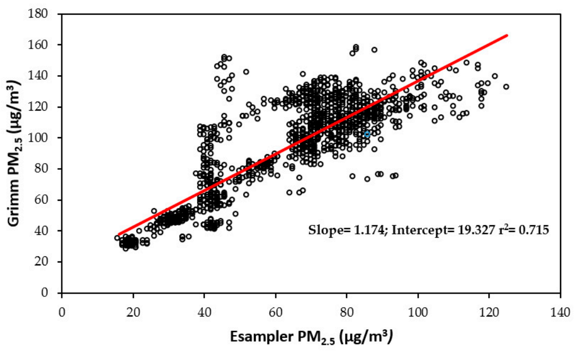

Figure S2: Comparison of data from Grimm and E-Sampler (used for indoor measurement)

Table S1: Correction factors of E-Samplers used for the study

E-Sampler	Slope	Intercept	R^2
Indoor	1.174	19.327	0.715
Outdoor	1.139	13.768	0.731
Simreni	0.936	16.608	0.713
Gathauli	1.325	19.851	0.736
Baghmara	0.886	15.113	0.739

S3: Description of microAeth and aethalometer; comparison between microAeth and aethalometer; description of Indoor Air Quality (IAQ) Probe

MicroAeth

Black carbon mass concentration was measured using the microAeth model [10]. The instrument is portable and siphons air at a constant flow rate of 50 mL/min. It measures the BC concentration based on the light absorption principle at the 880 nm wavelength of light. The inlet of the microAeth was fitted with a $PM_{2.5}$ cyclone in order to prevent particles with a larger diameter (greater than 2.5 micrometers) from getting inside the equipment. The microAeths used in the field were factory calibrated to ensure high-quality measurements.

Aethalometer

Aethalometer AE33 (Magee Scientific, USA) is a standard aerosol measuring equipment, used mainly for black carbon and brown carbon measurement. AE33 collects the air pollution sample and analyses it in seven optical wavelengths ranging from the near-infrared (950 nm) to the near-ultraviolet (370 nm) and provides real-time data. It measures the attenuation (ATN) of a light spectrum passing through a filter on which aerosols are continuously collected. Data obtained from the 880 nm channel is generally taken as black carbon reading.

Comparison of MicroAeth and Aethalometer

MicroAeths used in indoor and outdoor emission measurement were operated simultaneously with the aethalometer and the data obtained from all three equipment were compared. The scatter diagrams showing a comparison of data from the microAeth used in both indoor and outdoor smoke measurement are presented in **Figure S3** and the correction factors are given in **Table S2.**

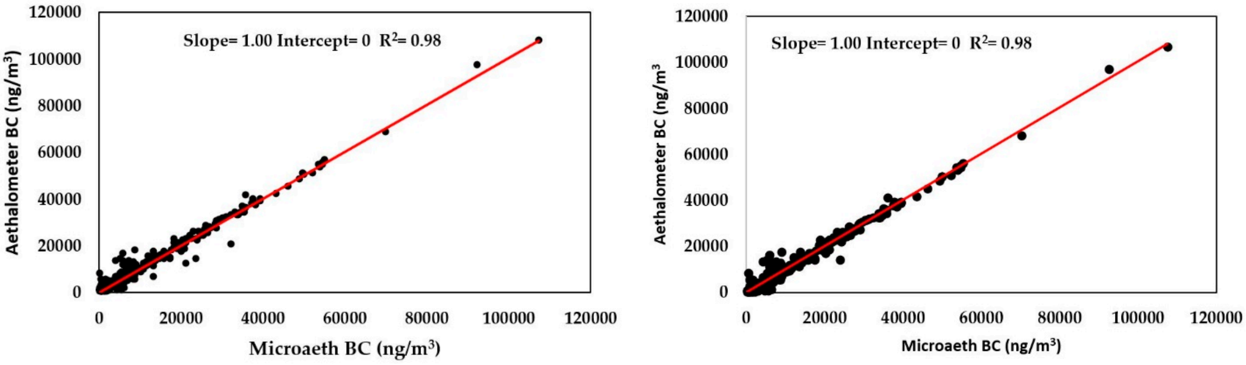

Figure S3: Comparison between aethalometer and microAeth measurements of indoor (left) and outdoor (right) emissions

Table S2: Correction factors of microAeth used for the study

MicroAeth	Slope	Intercept	R^2
Indoor	1.00	0	0.98
Outdoor	1.00	0	0.99

Indoor Air Quality (IAQ) Probe

The IAQ Probe was used to measure the CO_2 and CO concentration. It is a widely used, reliable equipment for measuring indoor air quality[11,12]. These probes were also compared against high-quality calibration gas standards (Specialty Gases Ltd. and Alchemic Gases and Chemical Pvt. Ltd., Mumbai, India) and subsequently correction factors were derived. The correction factor of the CO_2 measurement was calculated by comparing the data from sensor against standard CO_2 calibration

gas mixture concentration at 350 ppm and 1200 ppm (Specialty Gases Ltd.). Similarly the correction factor for CO sensor was also calculated by comparing the data from sensor against 5 ppm (Specialty Gases Ltd.) and 81 ppm (Alchemic Gases and Chemical Pvt. Ltd., Mumbai, India) CO calibration gas mixture.

S4: Emission factor calculation equation,

The total carbon (TC) in the cookstove smoke was calculated by the summation of carbon content in CO_2 (cCO_2), CO (cCO), BC. The emission factor (EF) of CO (EF_{CO}) was then computed as in Eqn (1):

$$EF_{CO}(gkg^{-1}) = \frac{cCO}{TC} \times F_c \times mCO \times 1000 \qquad (1)$$

Where Fc is the carbon weight fraction taken as 0.5 and mCO is the carbon fraction in CO. The computed EF was expressed in grams of pollutant emitted per unit kg of fuel wood combusted.

Similarly, the EF for CO_2 was computed as in Eqn 2:

$$EF_{CO2}(gkg^{-1}) = \frac{cCO_2}{TC} \times F_c \times mCO2 \times 1000 \qquad (2)$$

The EF for particulate pollutants such as $PM_{2.5}$ and BC were measured relative to EFCO following Eqn 3:

$$EF_x = \frac{Mx}{cCO} \times EFco \qquad (3)$$

Where, Mx is the mass concentration in $\mu g/m^3$ of particulate pollutants such as $PM_{2.5}$ and BC.

S5. Real-time emission factor plot for the measured kitchens

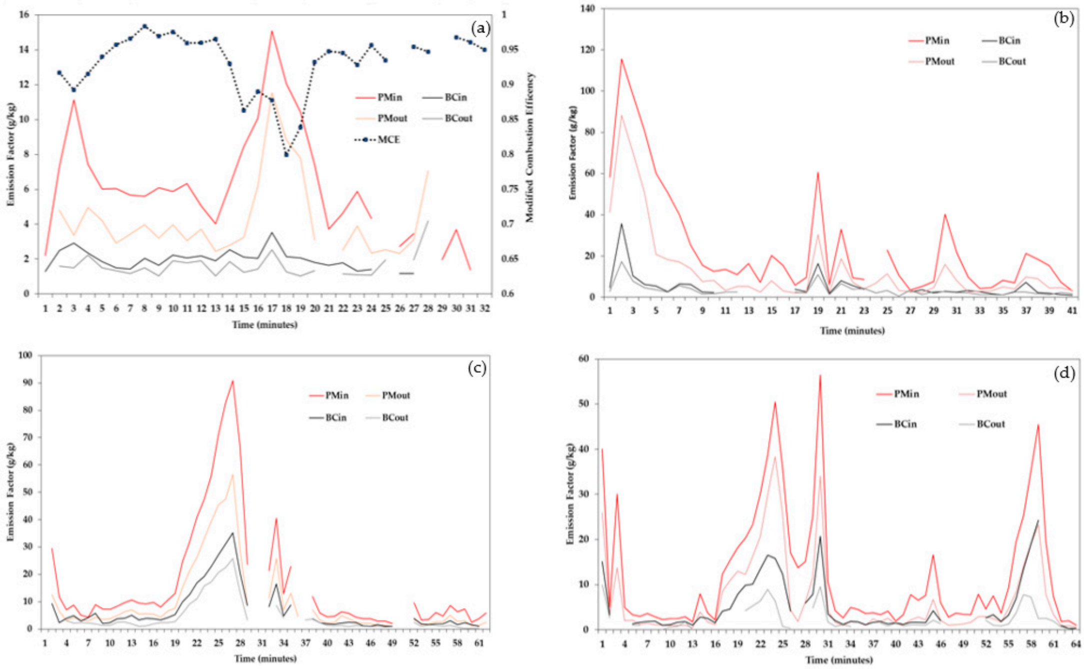

Figure S5: Figure showing real-time emission factor of $PM_{2.5}$ and BC for all kitchens indoor and outdoor along with real time indoor MCE for Kitchen 1.

S6: Daily average PM2.5 plot of both the villages

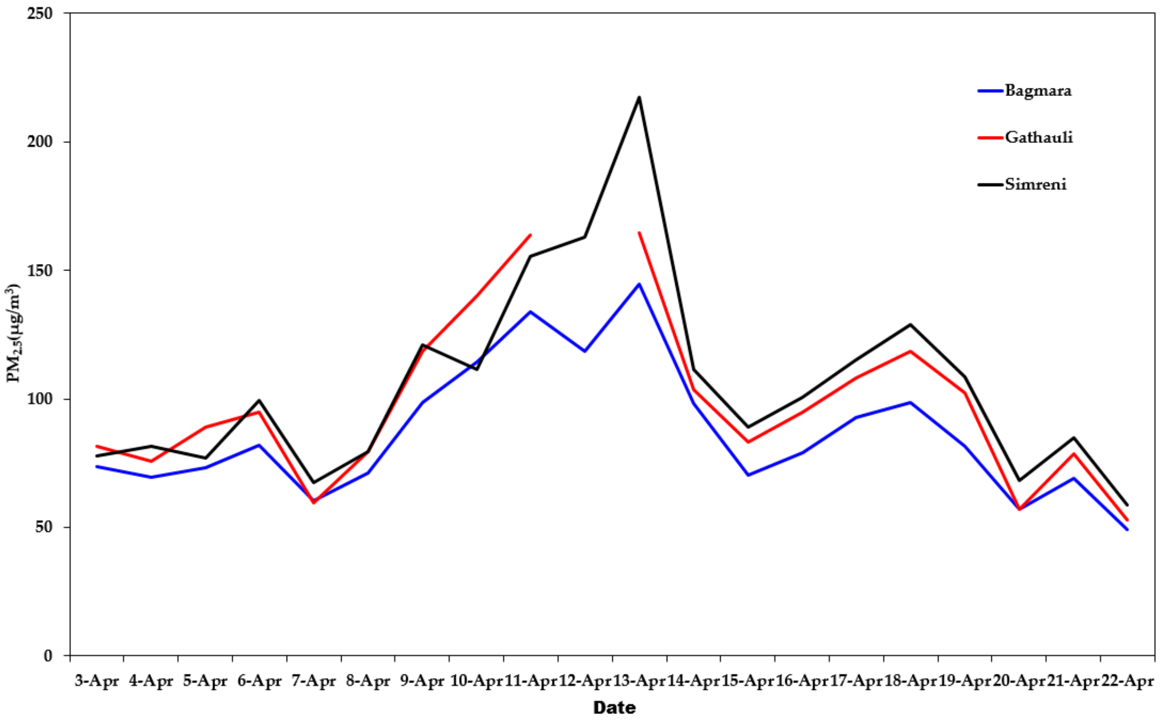

Figure S6: Daily average plot of all three ambient sites

S7. Meteorological parameters along with PM2.5

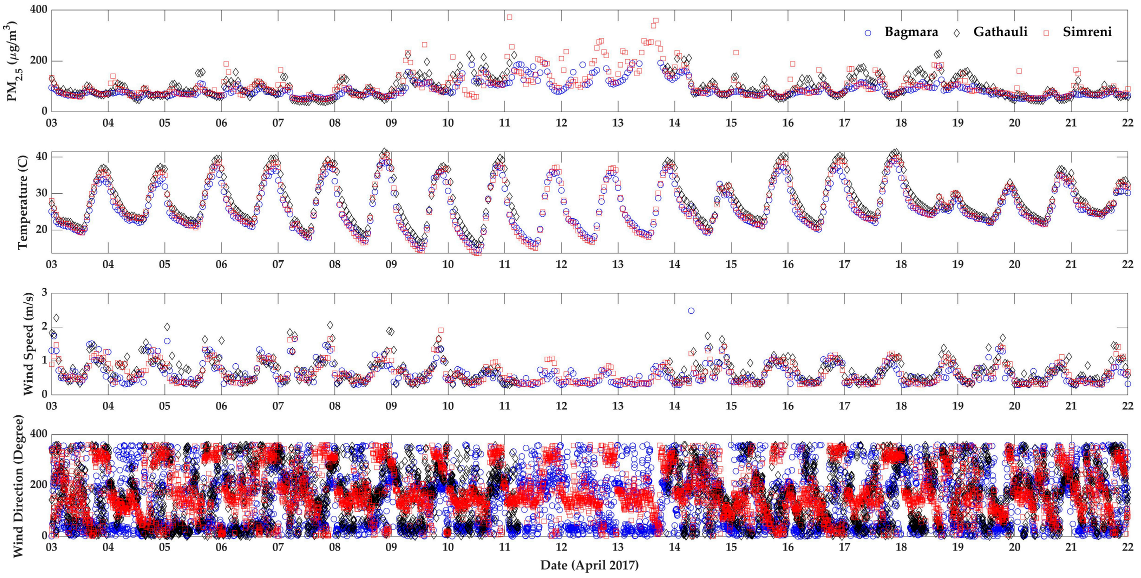

Figure S7: Real time measurement of (from top to bottom) ambient PM2.5 (μg/m3), temperature (°C), wind speed (m/s) and wind direction (degree) for all of the measured location

S8. Active fire images from MODIS

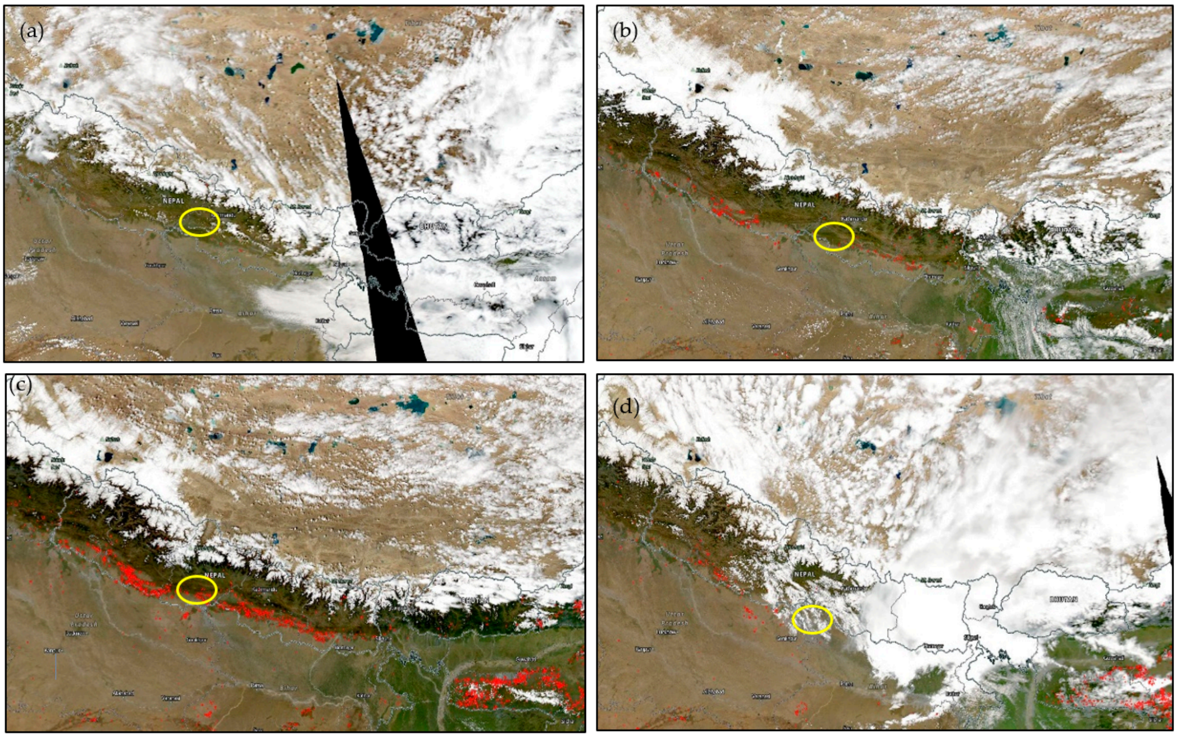

Figure S8: MODIS active fire images for (a) 3rd April (b) 8th April (c) 12th April and (d) 15th April 2017. The yellow circles in each of the map covers the study site

Reference

1. Mahapatra, P. S.; Jain, S.; Shrestha, S.; Senapati, S.; Puppala, S. P. Ambient endotoxin in PM 10 and association with inflammatory activity, air pollutants, and meteorology, in Chitwan, Nepal. *Sci. Total Environ.* **2018**, *618*, 1331–1342, doi:10.1016/j.scitotenv.2017.09.249.

2. Borlina, C. S.; Rennó, N. O. The impact of a severe drought on dust lifting in California's Owens lake area. *Sci. Rep.* **2017**, *7*, 6–9, doi:10.1038/s41598-017-01829-7.

3. O'Keeffe, D.; Dennekamp, M.; Straney, L.; Mazhar, M.; O'Dwyer, T.; Haikerwal, A.; Reisen, F.; Abramson, M. J.; Johnston, F. Health effects of smoke from planned burns: a study protocol. *BMC Public Health* **2016**, *16*, 1–7, doi:10.1186/s12889-016-2862-y.

4. Dominick, D.; Wilson, S. R.; Paton-Walsh, C.; Humphries, R.; Guérette, E. A.; Keywood, M.; Kubistin, D.; Marwick, B. Characteristics of airborne particle number size distributions in a coastal-urban environment. *Atmos. Environ.* **2018**, *186*, 256–265, doi:10.1016/j.atmosenv.2018.05.031.

5. Watson, J. G.; Chow, J. C.; Chen, L.; Wang, X.; Merrifield, T. M.; Fine, P. M.; Barker, K. Measurement system evaluation for upwind/downwind sampling of fugitive dust emissions. *Aerosol Air Qual. Res.* **2011**, *11*, 331–350, doi:10.4209/aaqr.2011.03.0028.

6. Mehra, M.; Panday, A. K.; Puppala, S. P.; Sapkota, V.; Adhikary, B.; Pokheral, C. P.; Ram, K. Impact of local and regional emission sources on air quality in foothills of the Himalaya during spring 2016: An observation, satellite and modeling perspective. *Atmos. Environ.* **2019**, *216*, 116897, doi:10.1016/j.atmosenv.2019.116897.

7. Wang, Y. Q.; Zhang, X. Y.; Sun, J. Y.; Zhang, X. C.; Che, H. Z.; Li, Y. Spatial and temporal variations of the concentrations of PM10, PM2.5 and PM1 in China. *Atmos. Chem. Phys.* **2015**,

15, 13585–13598, doi:10.5194/acp-15-13585-2015.

8. Chen, G.; Zhang, W.; Li, S.; Zhang, Y.; Williams, G.; Huxley, R.; Ren, H.; Cao, W.; Guo, Y. The impact of ambient fine particles on influenza transmission and the modification effects of temperature in China: A multi-city study. *Environ. Int.* **2017**, *98*, 82–88, doi:10.1016/j.envint.2016.10.004.

9. Guo, X.; Grimm, H.; Pesch, M.; Keck, L.; Spielvogel, J.; Schneider, F.; Brunnhuber, W. New methods for real-time measurement of environmental airborne particles. In *2010 4th International Conference on Bioinformatics and Biomedical Engineering, iCBBE 2010*; 2010; pp. 8–11.

10. Rehman, I. H.; Ahmed, T.; Praveen, P. S.; Kar, A.; Ramanathan, V. Black carbon emissions from biomass and fossil fuels in rural India. *Atmos. Chem. Phys.* **2011**, *11*, 7289–7299, doi:10.5194/acp-11-7289-2011.

11. Alves, C.; Duarte, M.; Ferreira, M. Air quality in a school with dampness and mould problems. *Air Qual. Atmos. Heal.* **2015**, *9*, 107–115, doi:10.1007/s11869-015-0319-6.

12. Vicente, E. D.; Ribeiro, J. P.; Custódio, D.; Alves, C. A. Assessment of the indoor air quality in copy centres at Aveiro, Portugal. *Air Qual. Atmos. Heal.* **2016**, *10*, 117–127, doi:10.1007/s11869-016-0401-8.
